# AgingPLUS: Theoretical Background and Study Design of the Randomized Trial

**DOI:** 10.1093/geroni/igab046.1681

**Published:** 2021-12-17

**Authors:** Manfred Diehl, George Rebok, David Roth, Kaigang Li, Abigail Nehrkorn-Bailey, Diana Rodriguez, Katherine Thompson

**Affiliations:** 1 Colorado State University, Fort Collins, Colorado, United States; 2 Johns Hopkins University, Baltimore, Maryland, United States

## Abstract

The AgingPLUS program targets three psychological mechanisms that are known barriers to middle-aged and older adults’ engagement in physical activity (PA): Negative views of Aging (NVOA), low self-efficacy beliefs, and poor goal planning skills. These risk factors are addressed in a 4-week intervention program that is compared to a generic health education program as the control group. Middle-aged and older adults (age 45-75 years) are enrolled in the trial for 8 months, with four assessment points: Baseline (pre-test), Week 4 (immediate post-test), Week 8 (delayed post-test), and Month 6 (long-term follow-up). The major outcome variables are participants’ engagement in PA as assessed via daily activity logs and actigraphs. Positive changes in NVOA, self-efficacy beliefs, and goal planning are the intervention targets and hypothesized mediating variables leading to increases in PA. This trial adopted the experimental medicine approach to assess the short- and long-term efficacy of the AgingPLUS program.

